# MicroRNA Zma-miR528 Versatile Regulation on Target mRNAs during Maize Somatic Embryogenesis

**DOI:** 10.3390/ijms22105310

**Published:** 2021-05-18

**Authors:** Eduardo Luján-Soto, Vasti T. Juárez-González, José L. Reyes, Tzvetanka D. Dinkova

**Affiliations:** 1 Departamento de Bioquímica, Facultad de Química, Universidad Nacional Autónoma de Mexico, CdMx 04510, Mexico; eduardolujan@quimica.unam.mx (E.L.-S.); vasti.juarez.gonzalez@gmail.com (V.T.J.-G.); 2 Departamento de Biología Molecular de Plantas, Instituto de Biotecnología, Universidad Nacional Autónoma de Mexico, Av. Universidad 2001, Cuernavaca Mor 62210, Mexico; jose.reyes@ibt.unam.mx

**Keywords:** maize, miRNA-target regulation, somatic embryogenesis, zma-miR528

## Abstract

MicroRNAs (miRNAs) are small non-coding RNAs that regulate the accumulation and translation of their target mRNAs through sequence complementarity. miRNAs have emerged as crucial regulators during maize somatic embryogenesis (SE) and plant regeneration. A monocot-specific miRNA, mainly accumulated during maize SE, is zma-miR528. While several targets have been described for this miRNA, the regulation has not been experimentally confirmed for the SE process. Here, we explored the accumulation of zma-miR528 and several predicted targets during embryogenic callus induction, proliferation, and plantlet regeneration using the maize cultivar VS-535. We confirmed the cleavage site for all tested zma-miR528 targets; however, *PLC1* showed very low levels of processing. The abundance of zma-miR528 slightly decreased in one month-induced callus compared to the immature embryo (IE) explant tissue. However, it displayed a significant increase in four-month sub-cultured callus, coincident with proliferation establishment. In callus-regenerated plantlets, zma-miR528 greatly decreased to levels below those observed in the initial explant. Three of the target transcripts (*MATE*, *bHLH*, and *SOD1a*) showed an inverse correlation with the miRNA abundance in total RNA samples at all stages. Using polysome fractionation, zma-miR528 was detected in the polysome fraction and exhibited an inverse distribution with the *PLC1* target, which was not observed at total RNA. Accordingly, we conclude that zma-miR528 regulates multiple target mRNAs during the SE process by promoting their degradation, translation inhibition or both.

## 1. Introduction

Among the distinctive features of plants, the capacity to generate an embryo by several routes is one of their most remarkable abilities. As evidence, in the late 1950s, carrot somatic cells exposed to a synthetic medium containing auxins and other phytohormones produced large numbers of embryos; this in vitro reprogramming, known as somatic embryogenesis (SE), was later found to be common in many plant species and could vary depending on many factors such as the plant genotype, the identity of the initial cells, and the usage of different combinations of growth regulators. Regardless of these singularities, SE can be used for the mass production of economically important plants, clonal propagation, germplasm conservation, protoplast culture, and genetic improvement of agronomic traits [1]. Additionally, SE has also emerged as a model system to study morphological, genetic, and molecular mechanisms shared with zygotic embryo development [2].

In some plants such as maize (*Zea mays*), somatic embryogenesis requires intermediary steps of induction and proliferation of the embryogenic callus before plant regeneration. The precise molecular mechanisms controlling these stages remain elusive. Over the last decade, small RNAs (sRNAs) have been identified as central regulators of the SE process in several plant species. Among plant sRNAs, microRNAs (miRNAs) remain the most studied and reported during SE. Plant miRNAs are 20–22-nt sRNAs transcribed from *MIR* genes by RNA polymerase II. Biogenesis enzymes successively process the primary transcripts into functional mature miRNAs that promote their complementary target silencing by cleavage or translational inhibition [3].

MiRNAs participate in SE by regulating the levels of many development-related transcription factors (TFs), components of hormone response pathways, and stress-response machinery found as essential for somatic to embryogenic transition in explant tissues. For example, in *Arabidopsis thaliana* SE induction, miR165 and miR166 target *PHABULOSA* (*PHB*) and *PHAVOLUTA* (*PHV*) mRNAs, encoding TFs that control the expression of a well-known SE marker and enhancer of the embryogenic potential *LEAFY COTYLEDON 1* (*LEC1*) [4,5]. Another miRNA involved in *Arabidopsis* SE is miR393, which targets *TRANSPORT INHIBITOR RESPONSE 1 (TIR1)*, encoding a receptor that participates in the auxin-regulated transcription of genes needed for proper SE induction [6]. In *Larix leptolepis*, overexpression of miR166a in somatic embryos resulted in the reduction of Class III homeodomain leucine zipper (HD-ZIP III) TFs that negatively impact the embryonic elongation and cotyledonary formation, leading to abnormal embryogenic development [7]; while miR156a overexpression in *Citrus sinensis* calli increased their embryogenic potential [8].

A substantial number of reports that have investigated the role of miRNAs (and other sRNAs) in SE are genome-wide approaches that describe significant changes in miRNA and mRNAs populations, but with no detailed experimental confirmation of their predicted targets and regulatory mechanisms during SE [9,10,11,12]. Previously, we globally examined the sRNA populations in immature embryos (IE) and derived embryogenic callus at different subculture times from the Mexican maize cultivar VS-535 [13]. We detected increases in abundance for several stress-related miRNAs (miR397, miR398, miR408, and miR528) toward the established proliferative stages of the callus. In particular, zma-miR528 was the most abundant miRNA in the explant (IE), with an initial decrement following induction and a further increase upon several subcultures. miR528 is restricted to monocots and was first described in rice [14,15]. Moreover, miR528 is one of the most abundant miRNAs in embryogenic calli for several plant species, including maize [15,16,17]. Multiple miR528 targets identified from different plant species have brought into focus the functional diversity of this miRNA [18]. A recent report showed that zma-miR528 affects lignin biosynthesis by targeting two copper-containing laccase transcripts, *ZmLACCASE3* (*ZmLAC3*) and *ZmLACCASE5* (*ZmLAC5*) under nitrogen luxury growth conditions (8 mM NO_3_^−^) in maize [19]. While an important number of targets have been predicted for this miRNA, only a few of them have been experimentally validated [16].

Here, we analyzed the accumulation patterns of zma-miR528 and some targets at total RNA level, and their polyribosomal distribution during SE of the maize cultivar VS-535. The abundance patterns of miR528 supported its relevance for target regulation at the proliferative stage of de-differentiated callus. The validated target transcripts encoding for *MATE*, *bHLH*, and *SOD1a* showed an inverse correspondence with the miRNA profile in total RNA samples. The inverse correlation for these targets was also evident in the independent process of maize seed imbibition and early germination. Importantly, miR528 was found at a higher proportion in the heavy polysome fractions in embryogenic callus and germinated embryo extracts, presenting an inverse correlation with the distribution of one putative target (*PLC1*), which did not present a negative correlation at total RNA level. All targets were validated for miR528-specific cleavage, suggesting that degradation is promoted for total or polysomal-bound transcripts. This study experimentally confirms several miR528 targets for maize SE and proposes versatile regulatory mechanisms for this miRNA at the RNA degradation and translation levels.

## 2. Results

### 2.1. Selection of Potential Targets Regulated by Zma-miR528

**Figure 1:**
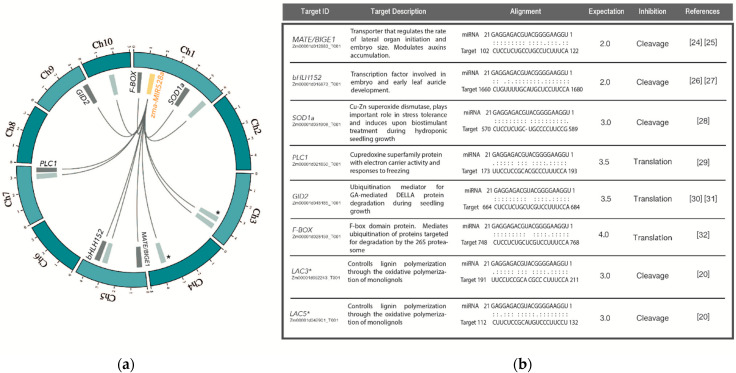
Target prediction for zma-miR528. (**a**) Circos plot between zma-miR528a and predicted targets showing their chromosomal location. The yellow-filled box represents the *MIR*528a gene location. Gray dark boxes refer to targets selected and analyzed in this study. For Circos plot construction, we used *zma-MIR528a* location only to illustrate the miRNA-target pairing, as both genes produce the same mature miRNA targeting the same transcripts. Pale gray boxes denote other putative targets not approached in our investigation. (*) Experimentally validated targets for zma-miR528 in a previous report: *ZmLAC3* (Zm00001d052243_T001); *ZmLAC5* (Zm00001d042901_T001). (**b**) List of the selected potential targets showing the recognition site pairing for zma-miR528 and their reported function in plant pathways according to several reports [19,23,24,25,26,27,28,29,30,31].

An initial approach to unveiling miRNA function is the identification of potential regulatory targets [10,20]. In maize, mature miR528 originates from two independent *MIR* genes, zma-*MIR*528a (MI0013240) and zma-*MIR*528b (MI0013239), located on chromosomes 1 and 9, respectively. Zma-miR528 mRNA targets were predicted using the psRNA Target program (http://plantgrn.noble.org/psRNATarget/, accessed on 23 February 2021) (Supplementary Materials, Additional File 1, Table S1) with the expectation threshold set at ≤ 4 to obtain a stringent search with high coverage [21]. The selection of targets for experimental validation was based on the appropriate miRNA-target pairing at the seed region (with penalization for G-U and other non-canonical pairings); a favorable secondary structure allowed access to the target site, and annotation of the target as a coding transcript [22] (see Supplementary Materials and Methods). Different transcripts with diverse functions and genomic locations were within the highly ranked targets (Figure 1a and Supplementary Material, Additional File 1, Table S1).

Novel putative targets selected for further experimental validation were *MULTIDRUG AND TOXIC COMPOUND EXTRUSION/BIG EMBRYO 1* (*MATE/BIGE1*; Zm00001d012883_T001), *BASIC HELIX-LOOP-HELIX 152* (*bHLH152*; Zm00001d016873_T001), *GIBBERELLIN INSENSITIVE DWARF2 F-BOX* (*GID2*; Zm00001d048185_T001), and a *F-BOX CONTAINING PROTEIN* (*F-BOX*; Zm00001d028159_T001). As well, we included the previously described targets *SUPEROXIDE DISMUTASE 1a* (*SOD1a*; Zm00001d031908_T001) and *PLASTOCYANIN-LIKE DOMAIN CONTAINING PROTEIN* (*PLC1*; Zm00001d021850_T001), the accumulation of which was previously reported during hormone depletion and regeneration of maize somatic embryos [17]. All selected targets seem to participate in pathways closely associated with the SE process, such as auxin accumulation and transport, oxidative-stress response, embryo development, gibberellic acid perception, and protein degradation process (Figure 1b). Notably, *LACASSE 3* (*ZmLAC3* (Zm00001d052243_T001)) and *LACASSE 5* (*ZmLAC5* (Zm00001d042901_T001)), previously validated and shown to impact on maize plant lodging under luxury nitrogen growth conditions [19], were also found in our search (Figure 1b; Supplementary Material, Additional File 1, Table S1).

### 2.2. Experimental Verification of miRNA-Guided Cleavage of miR528 Targets Using 5′-RLM-RACE

**Figure 2:**
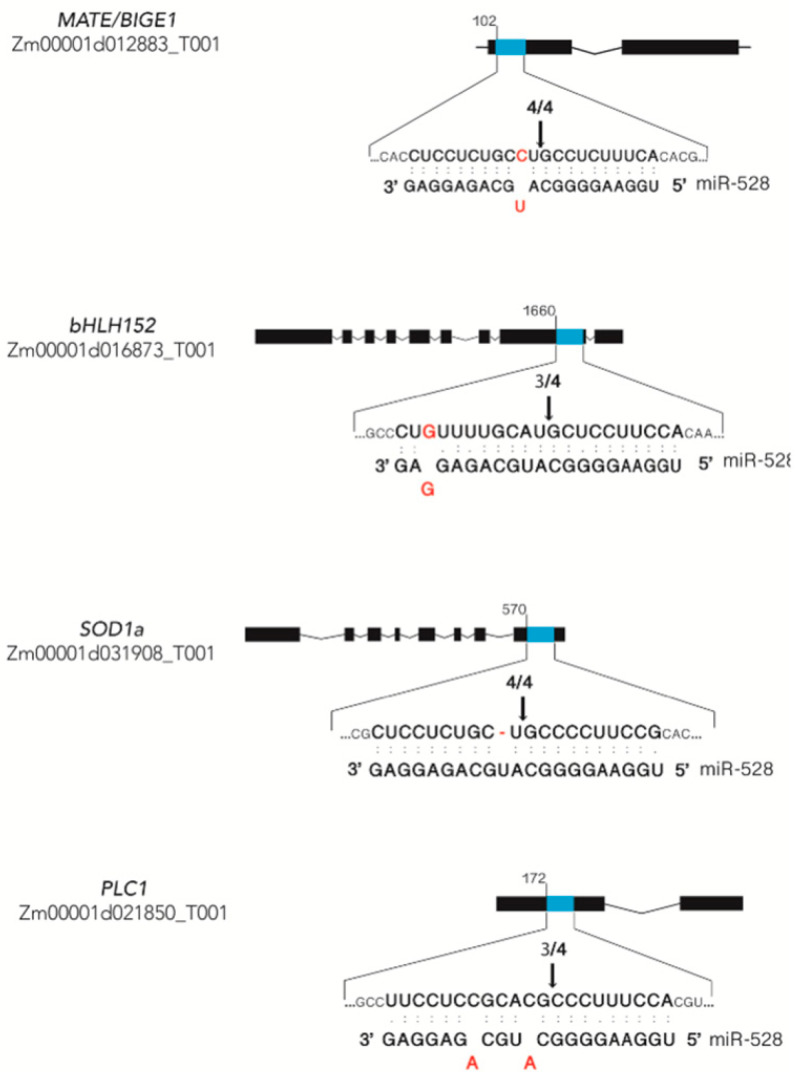
Experimentally validated cleavage sites for zma-miR528 targets. Validation for cleavage was obtained after 5′-RLM-RACE mapping, as described in the methods. The presence of cleavage products is shown in Supplementary Material, Figure S1. Arrows designate the cleavage sites, and the above numbers indicate the proportion of clones showing the same site. The upper strand represents targets with the complementary site (blue-filled fragment), and the bottom strand shows the pairing with the miRNA sequence. Mismatches are shown in red. (:) pairing, (.) non-canonical pairing.

To confirm whether zma-miR528 promotes cleavage of the selected targets, we performed 5′-RLM-RACE amplification and mapped the cleavage sites in the corresponding transcripts. The analysis demonstrated that the cleavage sites of *MATE/BIGE1*, *bHLH152*, and *SOD1a* were at the nucleotide that pairs with the 10th and/or 11th nucleotide from the 5′-end of zma-miR528 (Figure 2). Moreover, smaller amounts of the miRNA-mediated cleavage product of *PLC1* were detected, which led us to think that this target might experience another mode of regulation additional to transcript cleavage (Supplementary Material, Figure S1). Although *GID2* and *F-BOX* cleavage products were not detected, we pursued further analysis to gain insight into their feasible regulation as guided by zma-miR528.

### 2.3. Expression Patterns of Zma-miR528 and Targets during Maize Somatic Embryogenesis

**Figure 3:**
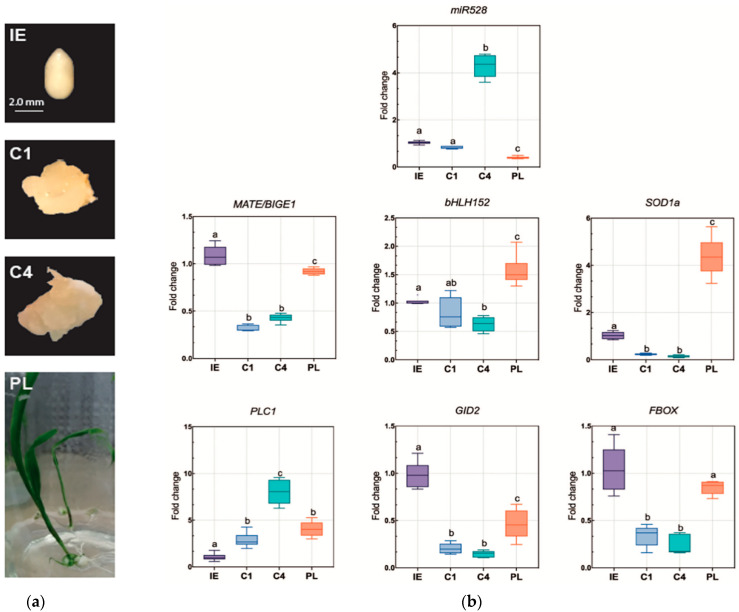
Zma-miR528 and mRNA target accumulation levels in VS-535 SE. (**a**) Different tissue samples obtained from maize SE stages used for miRNA and target profiling. Immature embryos (IE) at 15–18 days after pollination (DAP) used as initial explant for SE initiation. One month after induction, friable embryogenic callus (C1) was selected for sampling. Embryogenic calli were subcultured monthly, and proliferation was established after four months (C4). By gradual hormone reduction and photoperiod exposure, plantlet (PL) regeneration was achieved. (**b**) zma-miR528 and selected target levels were analyzed by RT-qPCR in total RNA from the samples mentioned above. Fold change represents abundance relative to IE and normalized either by U6 snRNA internal control (microRNA) or 18S rRNA (transcripts). The results were obtained from three independent biological replicates (*n* = 3) with three technical replicates for each one (*n* = 9). Data were analyzed by performing a one-way ANOVA with multiple comparisons by the Tukey post hoc test. Boxes that do not share at least an identical letter differ significantly (*p* < 0.005) from each other.

To better understand the zma-miR528 abundance fluctuation during SE stages, and if any of the selected transcripts exhibited a typical miRNA-target correspondence, we examined their accumulation patterns by RT-qPCR in immature embryos used as explants (IE), induced embryogenic callus of one month (C1), proliferating callus subcultured for four months (C4), and leaves of regenerated plantlets (PL) (Figure 3a). A slight, non-significant decrease in miR528 levels was observed for induced embryogenic callus (C1) compared to the original explant (IE) (Figure 3b); this could reflect the heterogenic nature of cells during calli induction and the remaining explant tissue that surrounds them. Indeed, a similar adjustment was reported for miR156, miR159, miR164, miR166, and miR319 families at early SE stages in *Arabidopsis thaliana* [9] and *Dimocarpus longan* [32]. However, in proliferating embryogenic calli (C4), miR528 importantly increased (~ almost 5-fold compared to previous stages, Figure 3b), consistent with our previous observation on a global analysis [13]. While miR528 remains highly accumulated at further subculture times (ten months after induction), a substantial reduction in abundance takes place in regenerated plantlets (PL), supporting its role in well-established embryogenic callus proliferation, but not in differentiated tissues, where a burst of development-related miRNA accumulation (miR156, miR160, miR164) has been described [17,33].

**Figure 4:**
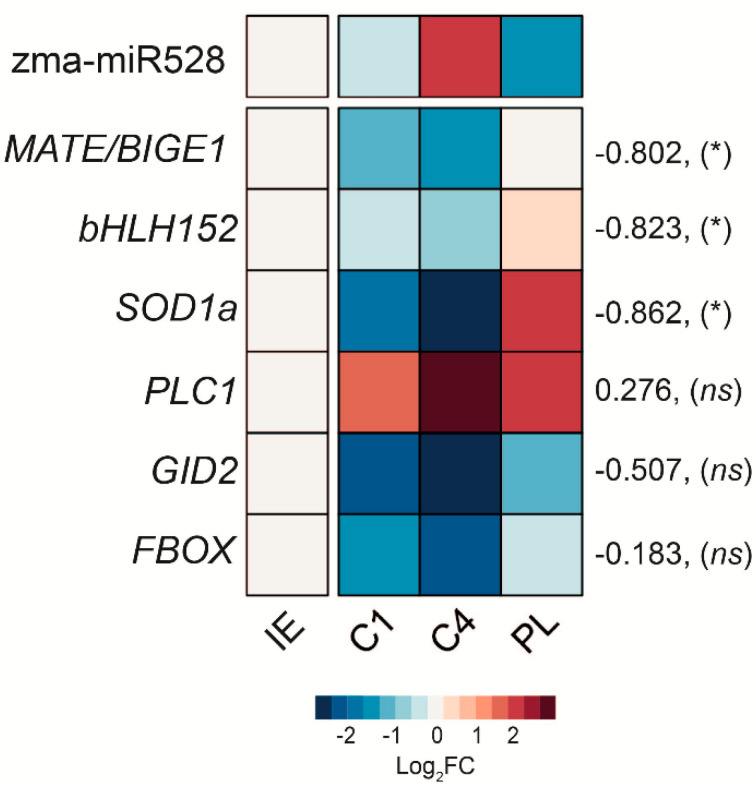
Inverse correlation between zma-miR528 and some of the selected targets during VS-535 SE stages. The heatmap shows the log2-fold-change (Log2FC) of each transcript in samples. Numbers on the right side represent Pearson’s coefficient (R) and the significance value (*p*) for the correlation analysis between the miRNA and each relative target abundance. * *p* < 0.05, *ns*: no significance. IE: immature embryo. C1: one-month embryogenic callus. C4: four-month embryogenic callus. PL: regenerated plantlet.

The quantification of selected zma-miR528 targets was performed in the same samples of RNA extraction. The abundance patterns of *MATE/BIGE1*, *bHLH152*, *GID2*, and *FBOX* presented some similarities at the assayed stages. All of them displayed significant downregulation during callus induction and in proliferating callus (C1 and C4), where miR528 was highly accumulated, but displayed upregulation in leaves of regenerated plantlets as miR528 decreased (Figure 3b). On the other hand, *PLC1* showed a divergent pattern compared to other targets, with constant increments from IE to C4 and a small but significant decrease in plantlets. The negative correlation between target transcript and zma-miR528 profiles, considering all analyzed tissues, showed significant values only for *MATE/BIGE1*, *bHLH152*, and *SOD1a* (Figure 4, Supplementary Material, Figure S2); which supports that degradation-mediated regulation of these targets by miR528 occurs during SE. *GID2* and *FBOX* transcripts tended to inverse correspondence only in C4 and PL, with poor statistical significance for a global inverse correlation (Supplementary Material, Figure S2). As expected, no correlation was obtained when comparing the miR528 pattern with *PLC1* accumulation during SE.

To test whether the observed correlations were also present in a different developmental model, we obtained expression profiles for zma-miR528 and the selected targets during dry seed imbibition and first stages of germination, a natural developmental program exhibiting variations in miRNA populations [34]. As in SE stages, *MATE/BIGE1, bHLH152*, and *SOD1a* presented similar accumulation patterns, indicating upregulation towards seedlings establishment concomitant with zma-miR528 level reduction (Supplementary material, Figure S3). Interestingly, the same profile was exhibited by *PLC1*, with increasing transcript accumulation after the first 24 h of imbibition. Most importantly, the previously determined strong inverse correlations for *MATE/BIGE1*, *bHLH152*, and *SOD1a* vs. miR528 were also present in the germination model, while *PLC1*, despite showing an inverse correlation, was not significant (Supplementary Material, Figures S3 and S4).

Overall, these findings suggest that miR528 exerts a versatile regulation over its targets, with some of them being preferentially regulated by degradation with clear inverse correlations, while others present only tendencies of inverse correspondence. Moreover, the absence of inverse correspondence between miR528 and some targets could imply that translation inhibition may be selected over cleavage as the prime regulatory mechanism, as prior evidence has pointed out [17]. Still, we cannot discard the possibility that the absence of correlation could stem from a lack of co-occurrence of the miRNA and targets in the same cell types of the analyzed tissues.

### 2.4. Distribution of Zma-miR528 and Its Targets in Polysome Profiles

**Figure 5:**
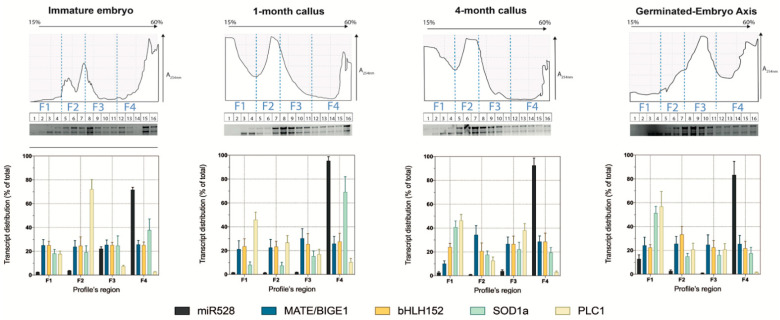
Distribution of zma-miR528 and targets in polysomal fractions. Upper panels show profiles for the immature embryo, one-month callus, four-month callus, and germinated embryo axis. Each profile is composed of 16 minor fractions further pooled into broader fractions according to the absorbance profile and rRNA observation on depicted agarose gels: F1 (free RNA and ribonucleoproteins complexes), F2 (monosomes), F3 (light polysomes), and F4 (heavy polysomes). Lower panels display the distribution of miR528 and some targets in each fraction. The percentage level was calculated for each transcript using the lower Ct value as a normalizer. Error bars indicate ± SD of two biological replicates with three technical replicates for each one (*n* = 6). A254 nm: absorbance value at 254 nm wavelength.

Inhibition of translation is a fundamental miRNA regulatory mechanism that recently gained importance in plants. Several key reports have provided evidence that translational inhibition is a plausible mode of regulation executed by plant miRNAs. For instance, some miRNAs exhibited disproportionate effects on their targets, when comparing the transcript levels with the corresponding protein abundance [35]. Furthermore, the association of miRNAs and miRNA-AGO1 complexes with polysomes (where active translation takes place) has been described [36,37,38]. Previously, we reported the high accumulation of miR528 in maize embryogenic calli polysomal fractions [17]. Based on the absence of inverse correlation for some targets in total RNA, and considering the zma-miR528 reported distribution in polysomal fractions, we evaluated the possible translational regulation by miR528 on some targets. (Figure 5).

The polysome profiles obtained on continuous sucrose gradients were divided into four main regions, representing distinct RNA pools. The F1 region was mainly composed of free RNA and small ribosomal subunit complexes; the F2 region was characterized by a well-defined monosome peak; the F3 comprised light and medium polysomes; the F4 region represented heavy polysomes. Polysomal profile fragmentation was made to evaluate miRNA and target transcripts distribution over complexes with different translation activities (Figure 5, upper panels). In all profiles, zma-miR528 accumulated mainly in the heavy polysomes region, more than 80% located in F4 for callus samples (Figure 5). Instead, *MATE/BIGE1*, *bHLH152*, and *SOD1a* targets were homogeneously distributed in all four regions of the profile, except for *SOD1a*, which for C1 had a higher abundance in F4. This suggests no inverse correspondence of the translational status of these targets with respect to the zma-miR528 location. In contrast, *PLC1* showed a unique distribution, as it preferably accumulated in free RNA or monosome regions (F1 and F2, respectively) and much less in F4 (10% or less), which inversely mirrored zma-miR528 distribution. The lower amount of *PLC1* detected in polysomes could be due to an initial translation inhibition triggered by zma-miR528 in polysomal complexes, which subsequently derivates in target cleavage and processing, as suggested by the lower amplification of *PLC1* cleavage product in 5′-RLM-RACE, and the absence of negative correspondence between this target and zma-miR528 levels in total RNA. In consequence, we hypothesize that zma-miR528 could promote transcript cleavage and translation inhibition over its targets.

## 3. Discussion

Plant cell totipotency is a field with a long history of research. In vitro culture of somatic embryos became a powerful biotechnological tool for the propagation and genetic improvement of several plants, crops included. So far, distinct molecular, biochemical, and morphological mechanisms are required to establish and maintain proper SE. Among those pathways, the regulation exerted by miRNAs is essential for controlling key processes in SE [9,39,40]. Thus, different SE stages are characterized by the expression of specific miRNAs.

Previous reports highlighted some miRNA families changing their accumulation during SE of different maize cultivars [12,13,16]. Accordingly, we found that zma-miR528 considerably accumulated in 15-day-old immature embryos used as explants and decreased as SE induction occurred. However, a posterior ~5-fold induction occurred upon proliferation and establishment of an embryogenic callus during later subcultures. Similar accumulation profiles have been reported for other miRNAs, such as zma-miR167, zma-miR398, and zma-miR827, and ath-miR164a-c and ath-miR398a-c, during advanced induction of maize and *A. thaliana* SE, respectively [9,13]. Moreover, zma-miR528 has been reported as one of the highly accumulated miRNAs in long-term subcultures (up until ten months) of maize embryogenic callus, which might reflect the role of this and other miRNAs in maintaining the elevated proliferative state of healthy well-established embryogenic callus [12,13]. The upregulation of zma-miR528 could act as an oxidative stress response modulator (its original classification in plants such as *Oryza sativa*), as portions of the explant tissue experience mild oxidation before calli establishment [12,41].

**Figure 6:**
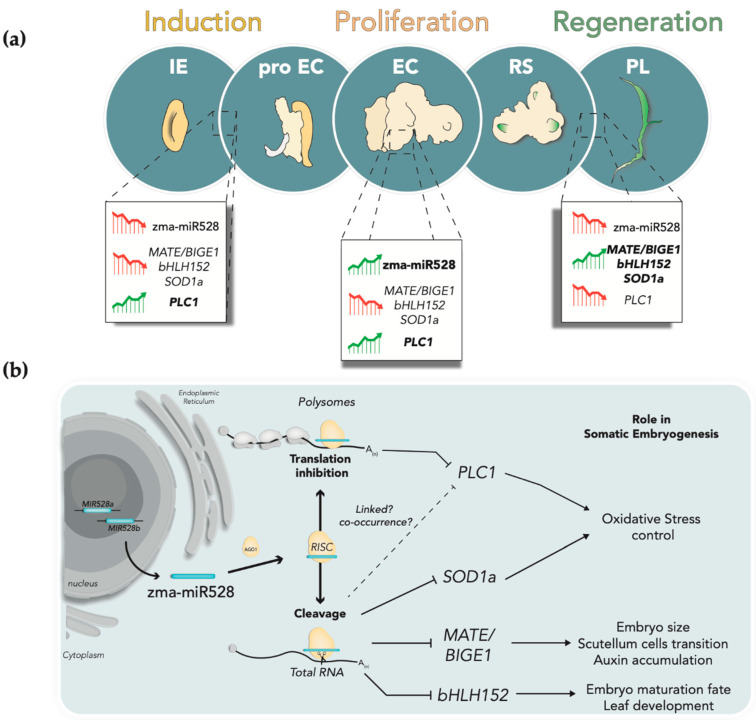
Mechanisms and impact of zma-miR528 target regulation during maize SE. (**a**) Zma-miR528 and its targets experience accumulation patterning during the induction, proliferation, and plantlet regeneration in maize SE. Upregulation: green upward arrow; downregulation: red downward arrow. IE: immature embryo (explant); proEC: pro embryogenic callus mass; EC: embryogenic callus; RS: regenerative spots; PL: regenerated plantlet. (**b**) Proposed model for zma-miR528 regulatory mechanisms exerted on targets. Mature miRNA arises from either of two *MIR528* genes present in maize. miRNA-RISC assembles either in the cytoplasm or on membrane-bound polysomes, where zma-miR528 directs target recognition to promote target cleavage or translational repression. Mechanism selection could depend on miRNA-target kinetic parameters or the presence/absence of certain accessory proteins in the RISC complex. However, either mechanism could be linked to the production of miRNA-mediated cleavage products, as suggested in the literature [38]. According to the target function, zma-miR528 could be involved in embryogenic calli proliferation by maintaining metabolic and physiologic processes required for SE.

The role of diverse miRNAs in almost any developmental plant process, including SE, has been approached by comparing the expression patterns of the mature miRNAs and their targets [1]. Particularly for zma-miR528, a wide range of potential targets exist, but few of them have been explored in detail. Here, we selected some of the predicted zma-miR528 targets, validated cleavage on a few of them and measured their transcript accumulation level during SE stages and seed imbibition. The zma-miR528-target inverse correlation or lack of it at particular stages, as well as the versatility of its regulatory mechanisms, expose the potential relevance of this maize miRNA, particularly enriched within the ES process (Figure 6). *MATE/BIGE1*, *bHLH152*, and *SOD1a* presented evident inverse correspondences with zma-miR528 levels in samples obtained from SE and early germination, which might imply that these targets are preferentially regulated by endonucleolytic cleavage. Indeed, miRNA-mediated cleavage products of these transcripts were easily detected when 5’-RLM-RACE was performed, consistent with the preferred plant miRNA mode of action and the significant repressive effect on target mRNA expression during developmental transitions and dedifferentiation processes [42].

Interestingly, *MATE/BIGE1* encodes a conserved transporter implicated in organ initiation and embryo size [23]. This loss-of-function mutation causes increased embryo size, early flowering, and accelerated lateral organ production. Moreover, *MATE/BIGE1* promotes an adequate transition to the expansion phase of scutellar cells during embryo development, a region from which maize embryogenic callus originated during the SE dedifferentiation step [33]. Thus, we propose that zma-mi528 might contribute to the delimitation of this region by controlling the abundance of *MATE/BIGE1* transcript to reach the callus establishment phase (Figure 6a). Additionally, *MATE/BIGE1* degradation promoted by zma-miR528 might stimulate auxin accumulation in proliferative embryogenic callus, as recent reports have confirmed that the overexpression of *ALTERED DEVELOPMENT PROGRAM 1 (ADP1)*, a *BIGE1* homologous gene in *A. thaliana*, causes a reduction in auxin level and diminishes its perception [43]. 

The functional characterization of several bHLH proteins in plants has been carried out mainly in *Arabidopsis* and rice (*Oryza sativa*), describing their roles in multiple cellular processes, such as embryo maturation, seed germination, flowering regulation, and stress response [44]. Furthermore, many *bHLH* factors are among the differentially expressed genes governing callus formation in maize SE [45]. According to our results, *bHLH152* was negatively regulated by zma-miR528 in well-established embryogenic calli until the regeneration of plantlets occurred. Similar downregulation of this target was observed during the early stages of SE using the inbred maize line CAL [45]. This TF has been associated with embryo maturation and early leaf development in maize [26]. The *Arabidopsis bHLH152* orthologue, *ROOTHAIRLESS LIKE-3 (LRL3)*/*bHLH82*, is principally involved in root-hair development [46]. During *Arabidopsis* SE induction, using the ecotype Col-0, the progressive reduction of *LRL3*/*bHLH82* level occurred as somatic embryos developed. In contrast, the steady expression of *LRL3/bHLH82* was observed when inducing SE from *tanmei/emb2757*, a mutant entirely lacking in vitro embryogenic response [47]. Therefore, the depletion of *bHLH152* levels by zma-miR528 might be involved in arresting the explant preestablished developmental pathway to allow dedifferentiation for SE (Figure 6b).

To date, several reports have supported the link between oxidative stress and SE [48]. Various authors consider H_2_O_2_ a cellular “second messenger” capable of inducing gene expression that leads to SE. It has also been shown that oxidative stress-inducing compounds promote endogenous auxin accumulation during the early dedifferentiation stage of SE in many plant species. Hence, a particular stress gradient is established in the explants during the cell dedifferentiation stage, guided by antioxidant enzymes scavenging reactive oxygen species that arise throughout the process [49,50]. In such a context, the downregulation of *SOD1a* caused by the zma-miR528 might cause the localized accumulation of H_2_O_2_ to promote specific expression programs and auxin accumulation that are crucial for embryogenic competence acquisition and maintenance during callus formation.

In agreement with our previous report [17], we corroborated a high accumulation of zma-miR528 in polysome fractions in all analyzed tissues. Several other functional miRNAs have been located in polysome fractions, providing evidence of translational control exerted by plant miRNAs [36,37]; the behavior of their targets has not been studied in such fractions. We hypothesized that some of the analyzed zma-miR528 targets might show opposite polysomal distribution between the SE stages, supporting translational control by the miRNA. Interestingly, *PLC1* was the only target that exhibited inverse distribution with zma-miR528 in polysomes, while the other targets roughly showed equal distribution across fractions. More notably, we could detect *PLC1* miRNA-mediated cleavage product, which may indicate that target excision occurs even when zma-miR528 is recruited to polysomes (Figure 6b). Interestingly, some reports have proposed that miRNA-guided degradation and translational arrest could be coupled in the cell, and the selection of the regulatory mechanism might depend on factors such as miRNA-target duplex subcellular location, recruitment of the mRNA transcript to polysomes, or assembly of suitable miRNA slicing machinery/complexes in aggregates that command the cleavage of transcripts [3]. This dual regulation has been proven for miR398, which mediates the cleavage and translational inhibition of mRNAs encoding COPPER CHAPERONE FOR SUPEROXIDE DISMUTASE 1 (CCS1), the chaperone protein that is essential for generating the mature copper/zinc SODs in *Arabidopsis* [51]. Several reports have demonstrated that translation inhibition by plant miRNAs preferentially takes place on membrane-bound polysomes (MBPs) assisted by ALTERED MERISTEM PROGRAM 1 (AMP1) and KATANIN 1 (KTN1) proteins [52]. Moreover, a recent report has revealed that target cleavage also occurs on MBPs, suggesting that both translational repression and endonucleolytic processing of miRNA targets co-occur at these subcellular domains [38]. Altogether, these findings might explain why we detected an inverse correspondence between zma-miR528 and *PLC1* only in polysome profiles. Correspondence between *PLC1* and miR528 levels was also reported. Li et al. found that this target, formerly known as *ANTI-FREEZE PROTEIN (AFP)*, did not display inverse correlation with zma-mir528, as the accumulation of the miRNA and *AFP* transcript both increased in the later stages of seed development [53]. Such unusual regulation exerted over *PLC1* during maize SE might be implicated in oxidative state control, since plastocyanins work as redox capacitors that can accept, store, and donate electrons during reactions catalyzed by the antioxidant enzymes [54], suggesting an interplay between PLC1 and the machinery controlling the oxidative gradient generation during SE.

Overall, this study addressed the differential regulation of one particular miRNA (zma-miR528) on diverse targets, and the plasticity of such control during the induction and proliferation of embryogenic calli within the maize SE process. Further research on the relevance of zma-miR528-mediated regulation on these and other targets in calli with distinct embryogenic potential, proliferative features and maize genotypes with contrasting embryogenic competence will contribute to a better comprehension of the role of miRNAs in SE, aiming at the improvement of in vitro plant propagation.

## 4. Materials and Methods

### 4.1. Plant Material and Tissue

All tissue samples were from the Mexican cultivar VS-535 *Tuxpeño* (*Zea mays* L.). Plants were grown in a greenhouse using potting sacks with a commercial soil mixture (Sunshine) from March to early July (considered to be spring in Mexico) under a natural photoperiod (approximately 12 h light/12 h dark) and temperatures between 25 °C and 30 °C, with watering three times per week. After four months, ears were manually pollinated, and similar-sized embryos were collected 15 days after pollination (IE) from the middle part of the ears. Embryos were used for somatic embryogenesis induction, morphological characterization, and RNA isolation. For imbibition, dry seeds were placed on wet cotton for 24, 48 and 72 h in the dark at 28 °C. The embryo axes were manually obtained from the imbibed seeds. After excision, embryonic axes were frozen immediately in liquid nitrogen until use.

### 4.2. Callus Induction, Subculture and Plant Regeneration

Callus induction and subculture were performed as previously reported [12,17,33]. Samples were collected one month (C1) and four months (C4) after induction. Every 2–3 weeks, the embryogenic calli were subcultured on fresh proliferation medium N6P, containing 2 mg L^−1^ 2,4-D and 0.1 mg mL^−1^ kinetin [55]. Regeneration took place using 8-month-old subcultures and followed the protocol reported by [55]. Segments of regenerated leaves were collected from the plantlets (PL) and immediately stored at −80 °C for subsequent analysis.

### 4.3. In-Silico Target Prediction for Zma-miR528

Zma-miR528 sequences were collected from the miRBase database (http://www.mirbase.org, release 22.1, accessed on 23 February 2021). The mature miRNA sequence UGGAAGGGGCAUGCAGAGGAG corresponds to either zma-miR528a-5p or zma-miR528b-5p and was used to identify target transcripts with the psRNATarget Server (V2, 2017 release, http://plantgrn.noble.org/psRNATarget/home; accessed on 23 February 2021). Default parameters were chosen and the Z. mays transcript, the NSF-funded Maize Genome Sequencing Project, Release 5a, filtered set was selected as cDNA library [56]. Target description was retrieved from the Maize Genetics and Genomics Database (maizeGDB RefGen_v4; https://www.maizegdb.org, accessed on 25 February 2021). Selection criteria of putative targets for experimental validation and expression analysis were: (1) strong miRNA-target pairing in the seed region; (2) expectation value ≤ 4 and appropriate target accessibility value (UPE < 0); and (3) annotation of the targets as known protein-coding transcripts [21,22].

### 4.4. Total and Polysomal RNA Isolation

Total RNA was isolated from IE, C1, C4, PL, or imbibed axes in at least triplicate biological samples following the protocol reported by [57]. Column-sized-fractionation, clean-up and concentration were performed using the RNA Clean and Concentrator TM-5 kit (Zymo Research) according to the manufacturer’s instructions. For polysome isolation and fractionation, nearly 1 g of each tissue was pulverized in liquid nitrogen with a sterile mortar and pestle. While still frozen, the powder was suspended in 10 mL of lysis buffer (200 mM Tris-HCl pH 8.5, 50 mM KCl, 25 mM MgCl2, 2 mM EGTA, 50 μg/mL cycloheximide, 0.5 mg/mL heparin, 0.5 μL/mL RNAsin, 50 μg/mL DTT, 1% μL Triton X-100), mixed by vortexing for 1 min and clarified by centrifugation at 15,000 rpm for 15 min at 4 °C. The supernatant was slowly layered onto 2 mL of sucrose cushion buffer (50 mM Tris-HCl pH 8.5, 25 mM KCl, 10 mM MgCl_2_, 60% sucrose and 0.05 mg/mL cycloheximide) in Ultra-Clear^®^ polycarbonate tubes (Beckman Coulter, Brea, CA, USA) and centrifuged at 50,000 rpm in a pre-chilled fixed angle 80Ti rotor for about 3 h, at 4 °C. The ribosomal pellet was resuspended in 500 µL of DEPC-treated sterile water, layered onto 15–60% continuous sucrose gradient, and centrifuged in a pre-chilled SW40 rotor at 36,000 rpm for 2 h, at 4 °C. Fractionation and profile plotting were performed with an Auto Densi-Flow system connected to an absorbance detector (wavelength 254 nm: ISCO-UV) and a 2-channel recorder (LKB 2210 Bromma). RNA was isolated from each fraction as previously reported [58]. RNA concentration was determined by Nanodrop and quality was tested by agarose gel electrophoresis.

### 4.5. Experimental Target Validation by RLM-RACE

Total RNA was obtained from germinated embryo axes and 3 to 5 µg of column-cleaned RNA were used as starting material for cDNA production, using FirstChoice RLM-RACE kit (Ambion, Austin, TX, USA) following the manufacturer’s directions, omitting the alkaline phosphatase and acid pyrophosphatase steps. After adapter ligation, PCR amplification of cDNA fragments was achieved by nested reactions using gene-specific primers designed for each target, based on their coding sequence reported in maizeGDB. Individual PCR fragments were cloned, and independent clones were sequenced to identify the amplified product and determine the cleavage site.

### 4.6. RT-qPCR

For large (>200 nt) RNAs, reverse transcription (RT) was performed using an oligo (dT) primer and the Improm-II^®^ reverse transcription system (Promega, Madison, WI, USA). Specific primers for each selected target were designed using Primer3Plus [59] with qPCR settings to amplify products containing the predicted miRNA-directed cleavage site (Supplementary material, Additional file 2, Table S2). For zma-miR528 amplification, stem-loop and forward primer were designed following previously reported recommendations [60]. Small RNAs (<200 nt) sample fractions were used in a pulsed stem-loop RT for zma-miR528 and U6 snRNA amplification. qPCR was performed using Maxima SYBR Green/ROX qPCR Master Mix in a 7500 Real-time PCR System (Applied Biosystems, Norwalk, CT, USA). Relative expression was calculated using the 2^−∆∆Ct^ method using IE (for SE samples) or dry (0 h) embryo axes (for imbibition stages) as references, and 18S rRNA (for targets) or U6 snRNA (for miRNA) as internal housekeeping controls [61]. Polysomal distribution was calculated as transcript abundance (% of total) along pooled fractions using the fraction with a lower Ct value as a reference. Results obtained for each condition were compared using a one-way analysis of variance (one-way ANOVA) with a Tukey Multiple Comparison post hoc test for significance.

## Supplementary Materials

The following are available online at https://www.mdpi.com/article/10.3390/ijms22105310/s1. Figure S1: gel image of 5′ RLM-RACE showing the amplified products (ranging from 100–200 bp) after consecutive PCR reactions, Figure S2: Pearson correlation analysis between zma-miR528 and the selected targets during SE, Figure S3: differential accumulation of zma-miR528 and target transcripts in dry (0 h) and imbibed (24–72h) embryonic axes, Figure S4: negative correlation between zma-miR528 and the selected targets abundances during maize seed imbibition. Table S1: putative targets for zma-miR528 identified using psRNATarget. Table S2: oligonucleotides used in this study.
