# Supplementary material for: MicroRNA Zma-miR528 Versatile Regulation on Target mRNAs during Maize Somatic Embryogenesis
Source: Int J Mol Sci. 2021 May 18;22(10):5310. doi: 10.3390/ijms22105310 (PMC8157881; doi:10.3390/ijms22105310)
Supplement: Supplementary file 1 [file ijms-22-05310-s001.zip › Lujan-Soto_etal_2021_Supplementary_figures.pdf]

Supplementary Figures

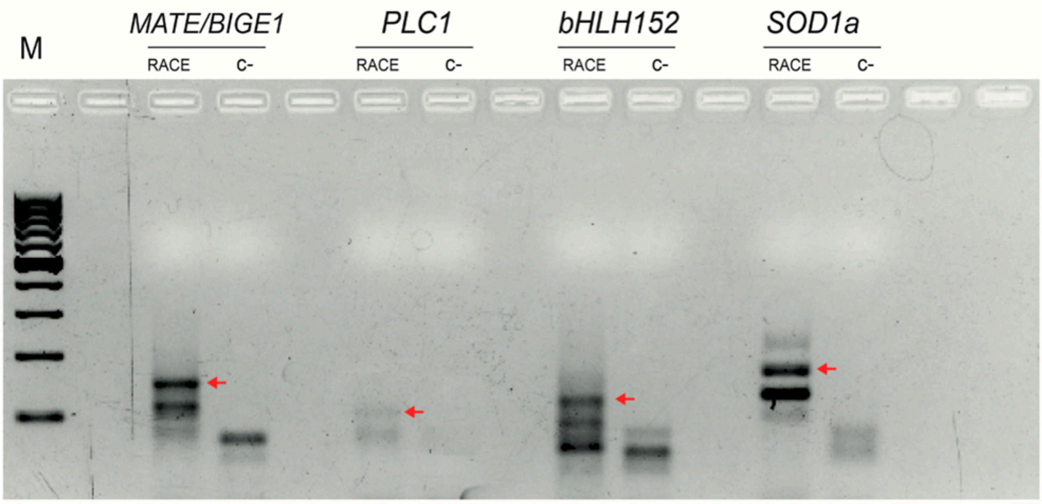

Figure S1. Gel image of 5’ RLM-RACE showing the amplified products (ranging from 100-200 bp) after consecutive PCR reactions. The slower migrating band in each lane (red arrows) was purified for DNA sequencing. C-: negative control for PCR.

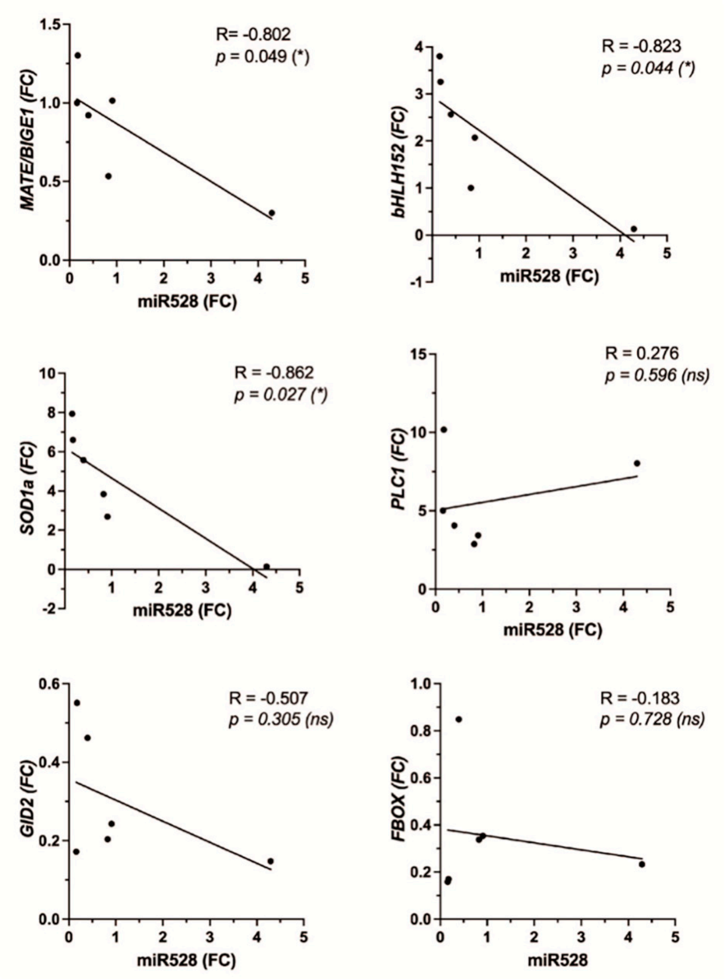

Figure S2. Pearson correlation analysis between zma-miR528 and the selected targets during SE. Pearson analysis was performed using the average fold change (FC) values in all tissues evaluated. Pearson correlations (R value) and statistical significance (*p* values) are displayed. * $p < 0.05$; ** $p < 0.01$, *ns*: no significance.

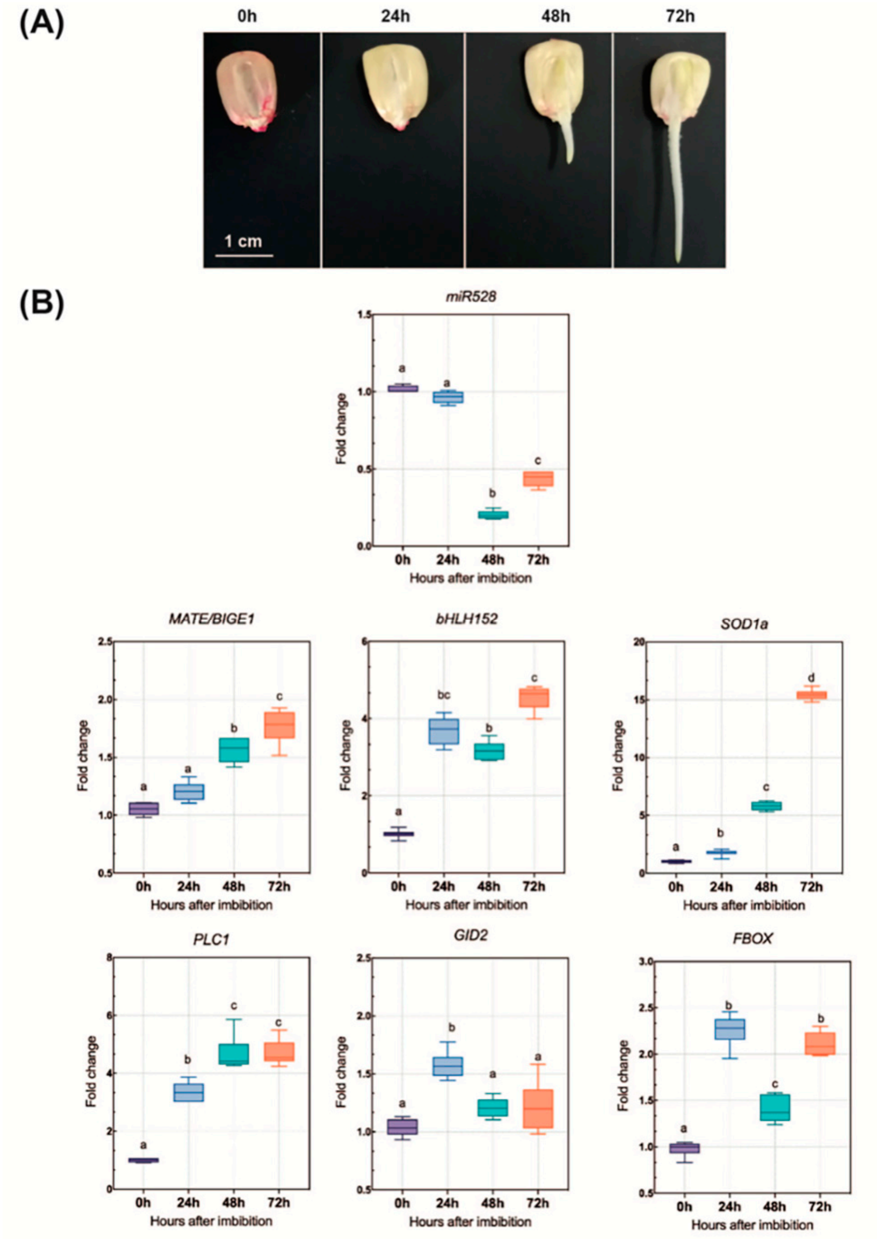

Figure S3. Differential accumulation of zma-miR528 and target transcripts in dry (0h) and imbibed (24h-72h) embryonic axes. (**A**) Representative images for the VS-535 maize seed from 0h to 72h of imbibition. (**B**) zma-miR528 and targets were analyzed by RT-qPCR at 0h, 24h, 48h, and 72h after imbibition. Fold change represents abundance relative to 0 h and normalized by U6 snRNA internal control for the microRNA or 18S rRNA for target transcripts. The results were obtained from three independent biological replicates (n= 3) with three technical replicates for each one (n=9). Data were analyzed by performing one-way ANOVA with multiple comparisons by the Tukey post-hoc test. Boxes that do not share at least a common letter differ significantly ($p < 0.005$).

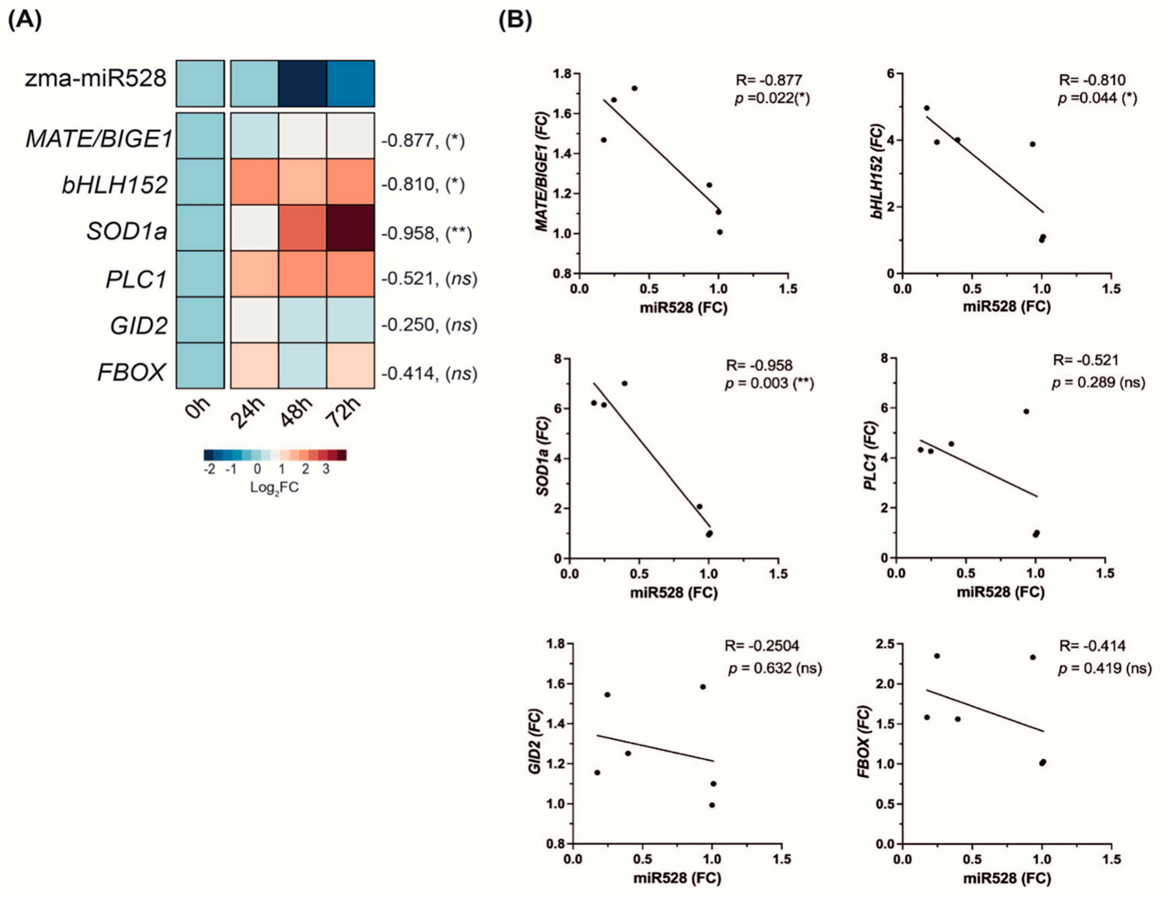

Figure S4. Negative correlation between zma-miR528 and the selected targets abundance during maize seed imbibition. (**A**) Heatmap showing log2-fold-change (Log_2FC) of zma-miR528 and selected targets. Numbers on the right side represent Pearson's coefficient (R) and the significance value (*p*) for the correlation analysis between zma-miR528 and each target relative abundances. (**B**) Pearson correlation analysis of fold changes between zma-miR528 and selected targets during imbibition and germination. Pearson correlations (R value) and statistical significance (*p* values) are displayed. * $p < 0.05$; ** $p < 0.01$, *ns*: no significance.
